# Neural–Cardiac Inflammasome Axis after Traumatic Brain Injury

**DOI:** 10.3390/ph16101382

**Published:** 2023-09-28

**Authors:** Robert W. Keane, Roey Hadad, Xavier O. Scott, Erika d. l. R. M. Cabrera Ranaldi, Jon Pérez-Bárcena, Juan Pablo de Rivero Vaccari

**Affiliations:** 1 Department of Neurological Surgery and The Miami Project to Cure Paralysis, University of Miami Miller School of Medicine, Miami, FL 33136, USA; rkeane@miami.edu (R.W.K.); edc74@miami.edu (E.d.l.R.M.C.R.); 2 Department of Physiology and Biophysics, University of Miami Miller School of Medicine, Miami, FL 33136, USA; 3 Intensive Care Department, Son Espases University Hospital, 07120 Palma de Mallorca, Spain

**Keywords:** inflammasome, traumatic brain injury, heart, caspase-1, ASC, inflammation

## Abstract

Traumatic brain injury (TBI) affects not only the brain but also peripheral organs like the heart and the lungs, which influences long-term outcomes. A heightened systemic inflammatory response is often induced after TBI, but the underlying pathomechanisms that contribute to co-morbidities remain poorly understood. Here, we investigated whether extracellular vehicles (EVs) containing inflammasome proteins are released after severe controlled cortical impact (CCI) in C57BL/6 mice and cause activation of inflammasomes in the heart that result in tissue damage. The atrium of injured mice at 3 days after TBI showed a significant increase in the levels of the inflammasome proteins AIM2, ASC, caspases-1, -8 and -11, whereas IL-1β was increased in the ventricles. Additionally, the injured cortex showed a significant increase in IL-1β, ASC, caspases-1, -8 and -11 and pyrin at 3 days after injury when compared to the sham. Serum-derived extracellular vesicles (EVs) from injured patients were characterized with nanoparticle tracking analysis and Ella Simple Plex and showed elevated levels of the inflammasome proteins caspase-1, ASC and IL-18. Mass spectrometry of serum-derived EVs from mice after TBI revealed a variety of complement- and cardiovascular-related signaling proteins. Moreover, adoptive transfer of serum-derived EVs from TBI patients resulted in inflammasome activation in cardiac cells in culture. Thus, TBI elicits inflammasome activation, primarily in the atrium, that is mediated, in part, by EVs that contain inflammasome- and complement-related signaling proteins that are released into serum and contribute to peripheral organ systemic inflammation, which increases inflammasome activation in the heart.

## 1. Introduction

Traumatic brain injury (TBI) results in disability and mortality, with survivors exhibiting extensive impairments in neurological function [1]. Increasing evidence reveals that TBI is not confined to the central nervous system (CNS), but has systemic effects that negatively affect the heart, lungs, liver, gut, thyroid, kidney, musculoskeletal system, and the immune system [2]. Systemic perturbations after TBI are induced as part of the secondary injury mechanisms, which include: neuroinflammation; dysregulation of the autonomic nervous system (ANS); activation of the hypothalamic–pituitary–adrenal (HPA) axis; and activation of the sympathetic nervous system (SNS), which result in the release of glucocorticoids and catecholamines, leading to systemic inflammatory effects in many organs following TBI [3,4,5,6].

TBI also results in the release of cytokines and chemokines into the systemic circulation, and we have previously shown that TBI induces inflammasome activation in the brain of mice subjected to severe TBI, which is associated with the development of acute lung injury [7]. Moreover, we proposed that following TBI, there is activation of a brain–lung axis, in which extracellular vesicles (EVs) secreted from the brain after trauma carry inflammasome proteins to the lungs and induce acute lung injury [8].

The inflammasome is a multiprotein complex involved in the innate immune response in the central nervous system (CNS) after injury events such as traumatic brain injury [9], spinal cord injury [10,11] and stroke [12,13], as well as after neurodegenerative diseases like Alzheimer’s disease [14], multiple sclerosis [15] and Parkinson’s disease [16]. The inflammasome is responsible for the activation of the inflammatory cysteine aspartase caspase-1 and the maturation of the inflammatory cytokine interleukin-1β [17], and inflammasome proteins have been shown to be present in EVs released after CNS injury in rodents and humans [7,18,19].

Non-neurologic organ dysfunction (N-NOD) occurs in approximately 80 to 90% of patients with TBI who are admitted to the intensive care unit, and it is associated with increased morbidity and mortality [20,21]. Neurogenic stunned myocardium (NSM) is a condition in which acute neurological events, such as TBI, give rise to cardiovascular complications, including increased troponin, increased atrial fibrillation, increased levels of cardiac enzymes, electrocardiogram (EKG) changes, hypertrophy, altered heart rate and left ventricular dysfunction [22]. A variety of mechanisms have been proposed to regulate post-traumatic cardiac dysfunction, which include: a heightened local and systemic inflammatory response; upregulation of the complement system; a surge of catecholamines; increase in oxidative/nitrosative stress; modifications of structural proteins; and alterations in calcium signaling [23]. However, the role of inflammasome activation in post-TBI cardiac dysfunction has not been clearly defined.

Here, we use a severe cortical impact model of TBI to study the inflammatory effects of TBI in the heart that are mediated by the inflammasome. We show that TBI induces cardiac inflammation characterized predominantly by myofibrillar degeneration and significant elevation of inflammasome proteins in the brain and heart of mice after TBI when compared to sham animals. Moreover, we characterize the protein content of serum-derived EVs in TBI patients and determine the effects of EV-containing inflammasome proteins on inflammation in cardiovascular cells in culture.

## 2. Results

### 2.1. Inflammasome Signaling Proteins Are Elevated in the Cortex after TBI

**Figure 1:**
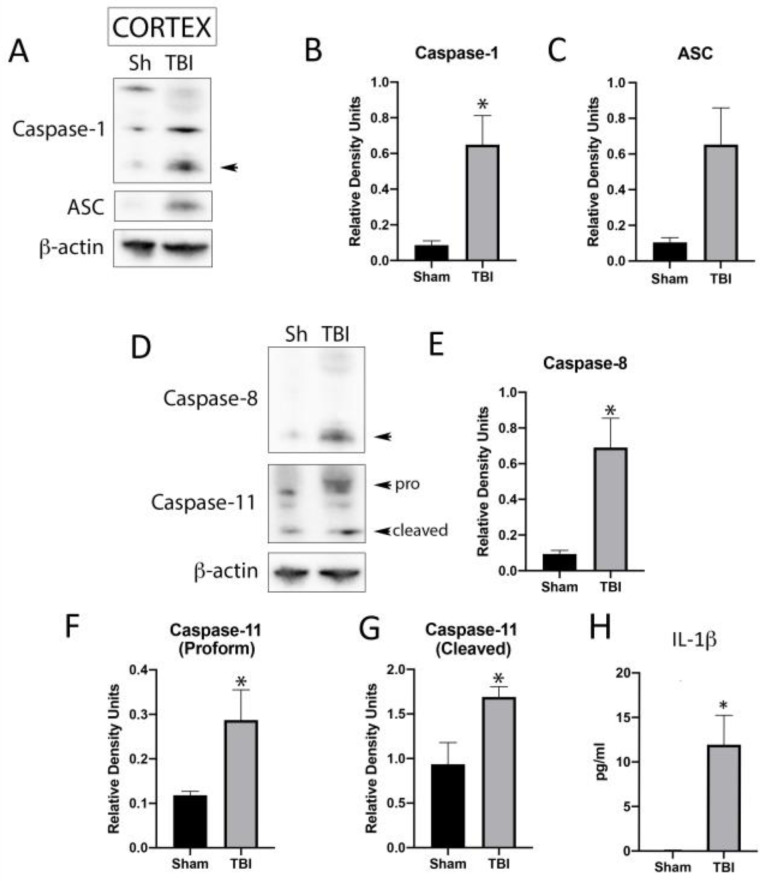
Inflammasome proteins in the cortex of TBI mice. Immunoblot of cortical protein lysates of sham and TBI mice that were injured and sacrifice 3 days after trauma and blotted for the canonical inflammasome proteins (**A**), caspase-1 (N = 7 per group) (**B**) and ASC (N = 6 per group) (**C**), as well as the non-canonical inflammasome proteins (**D**), caspase-8 (N = 7 per group) (**E**), caspase-11 (**F**): pro-form, (**G**): active form) (N = 7 per group) and IL-1β (N = 7 per group) (**H**). Arrow heads in caspase-1 and capase-8 correspond to the cleaved active form of the protein of interest. β-actin was used as a protein loading control and internal standard. Data presented as mean + SEM. * *p* < 0.05. Arrow in (A) points to bands that were quantified.

We have previously shown that the pro-inflammatory cytokine IL-1β, which is regulated by inflammasome activation, is elevated in the brain of mice after TBI [9]. Since the inflammasome is a major regulator of the innate immune response mediated by IL-1β after TBI [24,25], we aimed to use immunoblotting to determine the protein levels of several key inflammasome signaling components. Thus, protein lysates of sham and TBI mice were immunoblotted for canonical (Figure 1A) and non-canonical (Figure 1D) inflammasome protein expression. Accordingly, the protein levels of caspase-1 (Figure 1B), ASC (Figure 1C), caspase-8 (Figure 1E) as well as the pro- (Figure 1F) and cleaved (Figure 1G) forms of caspase-11 were statistically significantly elevated in TBI mice when compared to sham mice. We then performed an electrochemiluminescent immunoassay (ECLIA) for the downstream inflammasome signaling cytokine IL-1β in the brain of TBI mice that were sacrificed 3 days after injury using the Meso Scale Discovery platform with the MESO Quickplex SQ 120 instrument,(MSD, Rockville, MD, USA). Our findings indicate that the protein levels of IL-1β in the cortex (Figure 1H) were elevated in the tissues of brain-injured mice when compared to sham controls. These findings indicate that there is an increase in the inflammatory response present in the brain after TBI that is consistent with increased inflammation in the cortex of brain-injured mice.

### 2.2. Pyrin Is Elevated in the Brain of Mice after TBI

Several inflammasome sensors have been described to play a role in inflammasome signaling at different time points after TBI, such as the NLRP1, AIM2 and NLRP3 inflammasomes [9,26,27]. Here, we immunoblotted brain lysates from TBI and sham animals that were sacrificed at 3 days after TBI, for different inflammasome sensors (Supplementary Figure S1A). Of the inflammasome sensors that were analyzed (NRLP1, NLRP3, NLRC4, AIM2 and pyrin), pyrin was the only inflammasome sensor that was elevated at 3 days after TBI in the cortex of these mice (Supplementary Figure S1B), suggesting that the pyrin inflammasome may play a role in the neuroimmune response acutely after severe TBI in C57BL6 mice.

### 2.3. ASC Specks Are Elevated in the Brain of Mice after TBI

**Figure 2:**
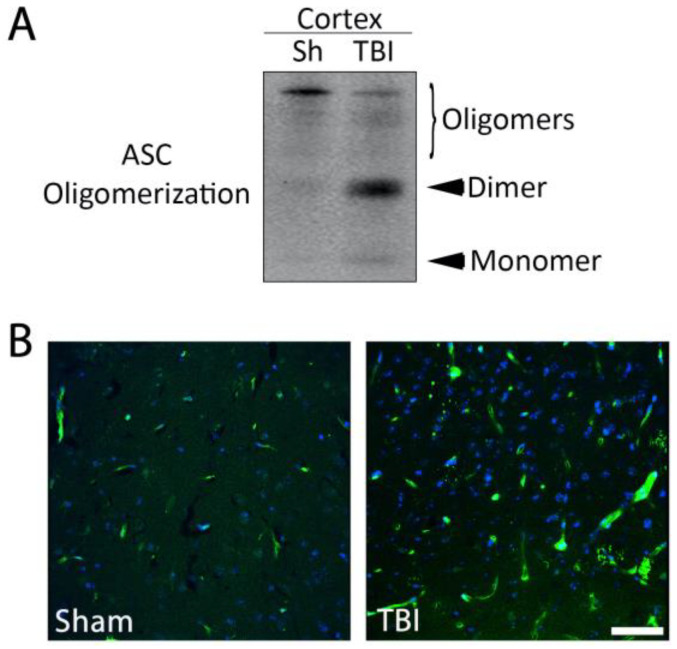
ASC specks in the cortex of sham and TBI mice. Brain cortices were harvested from sham and 3-day TBI animals, then processed with partial purification of the pyroptosome and resolved with immunoblotting for the expression of ASC monomers, dimers and oligomers (**A**). Brains were also collected after perfusion/fixation from sham and TBI ASC-citrine mice, and the cortex was imaged with confocal microscopy. ASC-citrine (green), DAPI (blue) (**B**). Bar graph = 25.06 μm.

Inflammasome activation induces ASC to form a speck in cells that leads to caspase-1 activation and pro-IL-1β processing. ASC specks are released into the extracellular space, where they continue to process pro-IL-1β even after cell death by pyroptosis [28]. Thus, to analyze ASC speck formation after TBI, we performed a partial purification of the pyroptosome to analyze ASC oligomerization in the cortex of sham and TBI animals (Figure 2A). ASC oligomerization was greater in the cortex of TBI animals when compared to sham animals (Figure 2A). In addition, we observed increased immunofluorescence of ASC specks after TBI in the cortex of ASC-citrine mice (Figure 2B), supporting the idea that TBI induces inflammasome activation in the brain (Figure 1C). Together, these data indicate that ASC expression increases in the brain after TBI and that increased ASC specks are present in the cortex of mice after TBI.

### 2.4. Inflammasome Signaling Proteins Are Elevated in the Heart after TBI

**Figure 3:**
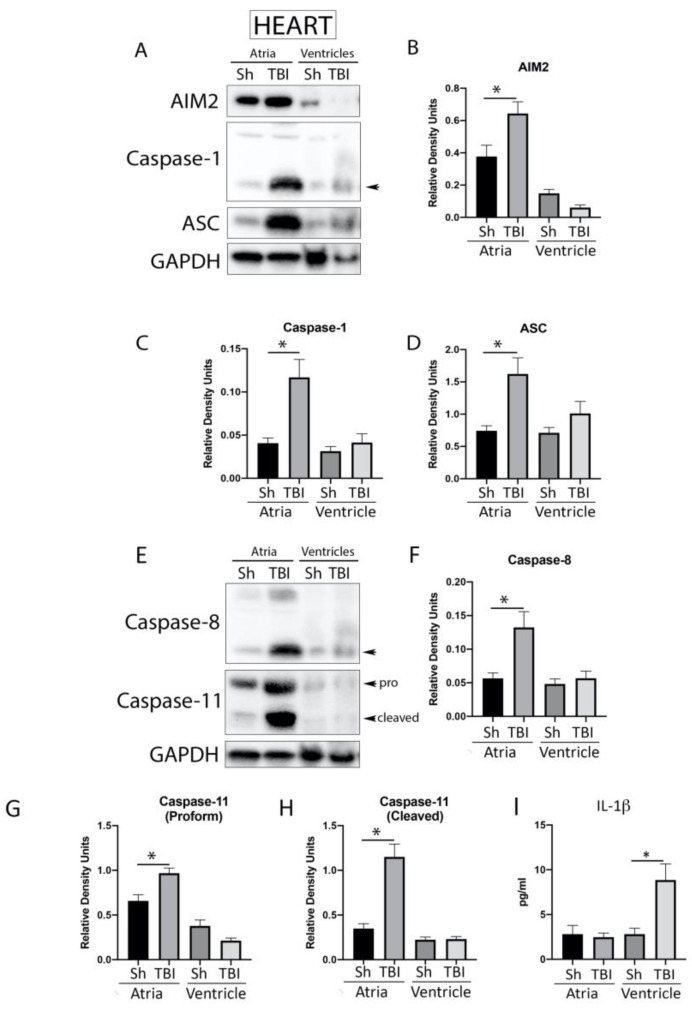
Inflammasome proteins in the heart of TBI mice. Immunoblot of heart protein lysates obtained from the atria and ventricles of sham and TBI mice that were injured and sacrifice 3 days after trauma and blotted for the canonical inflammasome proteins (**A**) AIM2 (N = 6 per group) (**B**), caspase-1 (N = 6 per group) (**C**) and ASC (N = 6 per group) (**D**), as well as the non-canonical inflammasome proteins (**E**) caspase-8 (N = 6 per group) (**F**), caspase-11 (N = 6 per group) (**G**): pro-form, (**H**): active form) and IL-1β (N = 6 per group) (**I**). Arrow heads in caspase-1 and capase-8 corresponds to the cleaved active form of the protein of interest. β-actin was used as a protein loading control and internal standard. Data presented as mean + SEM. * *p* < 0.05. Arrows point to bands that were quantified.

TBI causes a systemic inflammatory response, mediated by the inflammasome, that affects the lungs and is responsible for causing acute lung injury [7,28,29]. In addition, it has been previously shown that isolated diffuse cerebral injury may result in cardiac complications following TBI [30,31]. To determine the protein levels of canonical (Figure 3A) and non-canonical (Figure 3E) inflammasome signaling proteins in the atria and ventricles of sham and 3-day TBI mice, atria and ventricle protein lysates were immunoblotted for AIM2 (Figure 3B), caspase-1 (Figure 3C), ASC (Figure 3D), caspase-8 (Figure 3F) and caspase-11 (Figure 3G, H). Quantification of band densities indicated a significant increase in the atria of brain-injured mice for all these proteins when compared to sham animals. However, we did not detect a significant increase in these proteins in the ventricles following injury, indicating that TBI activates the inflammasome complex mainly in the atria. In addition, to determine if TBI also induces an inflammatory response in the heart after TBI, we performed a protein analysis assay for the downstream inflammasome signaling cytokine IL-1β in the heart of TBI mice that were sacrificed 3 days after injury. Our findings indicate that the protein levels of IL-1β in the heart (Figure 3I) were elevated in the tissues of brain-injured mice when compared to sham controls. Interestingly, the protein levels of IL-1β were only elevated in the ventricles of TBI mice when compared to sham mice, whereas the levels of IL-1β in the atria remained unchanged (Figure 3I).

### 2.5. TBI Induces Histological Myocardial Abnormalities

**Figure 4:**
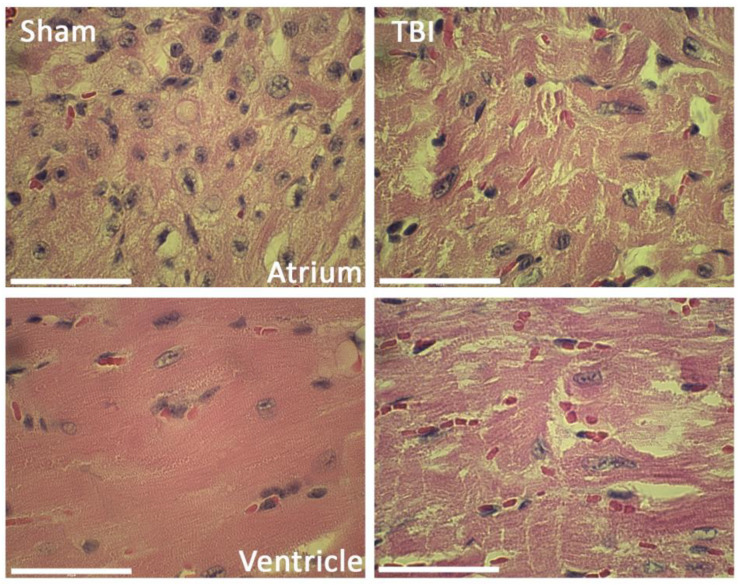
H&E and Masson’s *Trichrome* staining of the heart of sham and TBI mice. Representative light microscopy images of paraffin-embedded sections corresponding to the atrium and ventricle of sham and TBI mice that were sacrificed 3 days after TBI and stained with H&E. Myofibrillary degeneration (myocytolysis) is evident as affected fibers with altered cross-striations. Scale bar: 50 μm.

To determine whether severe CCI induced alterations in cardiac myofibrillary cytoarchitecture, paraffin-embedded heart sections were stained with hematoxylin and eosin (H&E) (Figure 4). Cross-striations within myocardial muscle cells were affected in TBI animals, with altered transverse bands in the sarcoplasm when compared to heart sections from sham animals, suggestive of myocardial contraction band necrosis. Therefore, acute neurological events such as TBI give rise to cardiovascular abnormalities, including myofibrillary degeneration or myocytolysis [22,32].

### 2.6. ASC Specks Are Elevated in the Heart of Mice after TBI

**Figure 5:**
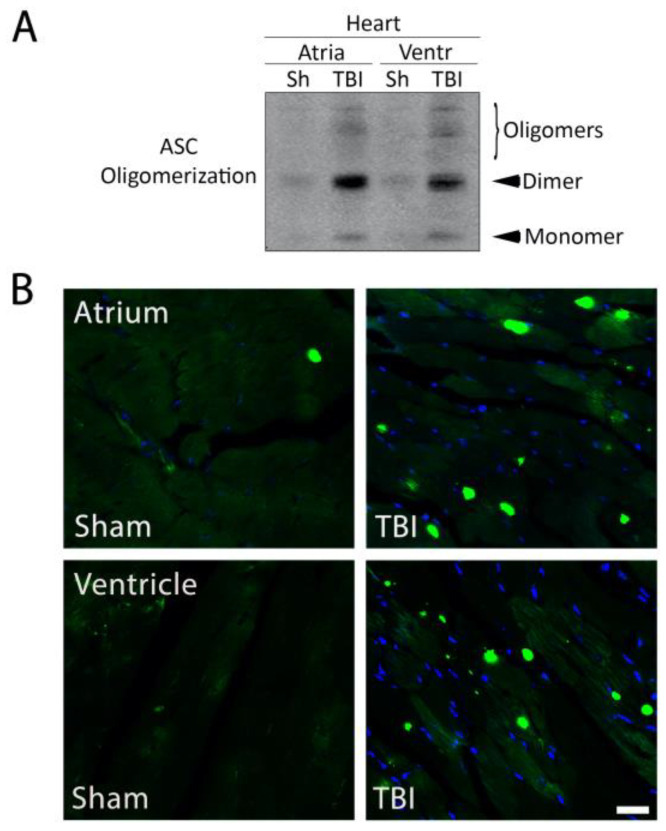
ASC specks in the heart of sham and TBI mice. Hearts were harvested from sham and 3-day TBI animals, processed with partial purification of the pyroptosome and resolved with immunoblotting for the expression of ASC monomers, dimers and oligomers (**A**). Hearts were also collected after perfusion/fixation from sham and TBI ASC-citrine mice, and the atrium and ventricle were imaged with confocal microscopy. ASC-citrine (green), DAPI (blue) (**B**). Bar graph = 15.91 μm.

To establish whether TBI induced systemic alterations in ASC expression in the heart, we analyzed ASC oligomerization and performed immunohistochemical analysis of ASC specks in heart sections from ASC-citrine mice subjected to severe TBI. As shown in Figure 5, TBI resulted in increased ASC oligomerization in the heart of TBI mice (Figure 5A) and induced ASC speck formation in the heart when compared to sham mice (Figure 5B), indicating that TBI causes increased inflammasome activation and ASC specks in the heart.

### 2.7. Serum-Derived EV Characterization after TBI

**Figure 6:**
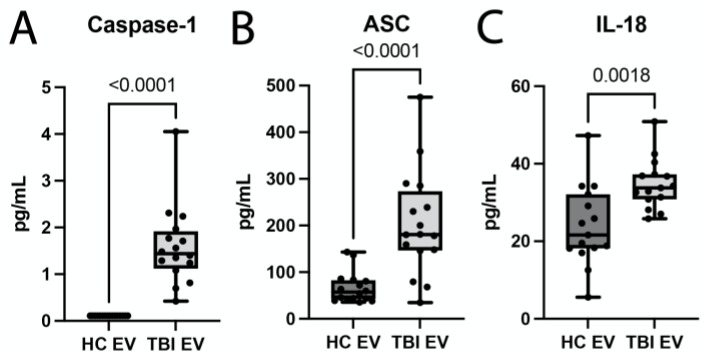
Inflammasome proteins in serum-derived EVs from TBI patients. Expression of inflammasome proteins caspase-1 (N = 16 per group) (**A**), ASC (N = 16 per group) (**B**) and IL-18 (N = 15 per group) (**C**) in EVs from TBI patients and healthy controls (HC). Data presented as min and max.

We have previously shown that inflammasome proteins are present in EVs, and that these EVs are carried throughout the body, inducing an inflammatory response in the lungs after TBI [7,18,28,29]. Consistent with previous studies, here, we isolated EVs from the serum of TBI patients and healthy controls (HCs) and characterized them for particle size (Figure S2A) and particle number (Figure S2B), as well as the expression of the inflammasome signaling proteins caspase-1 (Figure 6A), ASC (Figure 6B) and IL-18 (Figure 6C), along with the EV marker CD63 (Figure S2C). Accordingly, we found that EV size in HCs was approximately 181 nm, and it was 175 nm in TBI patients (Figure S2A). In addition, there was also a significant increase in EV number isolated from the serum of TBI patients when compared to HCs (Figure S2B). Furthermore, the protein levels of the inflammasome signaling proteins caspase-1 (Figure 6A), ASC (Figure 6B) and IL-18 (Figure 6C), as well as the EV marker CD63 (Figure S2C), were elevated in serum-derived EVs from TBI patients when compared to HCs.

We then carried out a mass spectrometry analysis of isolated EVs from TBI mice (3 days after TBI) and HCs and found that there was an overlap of 72 protein between the sham and TBI mice. However, there were a distinct group of 5 proteins (fibrinogen beta chain, Ig heavy chain V region J539, keratin type II cytoskeletal 2 epidermal, fibrinogen alpha chain, Ig kappa chain V-V region L6 (fragment)) in the sham group and 10 proteins (inter-alpha trypsin inhibitor heavy chain H3, histidine-rich glycoprotein, fetuin-B, Ig kappa chain V-III region, alpha-2-antiplasmin, glutathione peroxidase 3, Ig kappa chain V-III region PC 3741/TEPC III) in the TBI group that did not overlap with the sham group (Figure S3A). Moreover, of the proteins analyzed, we found that complement C3, the serine protease inhibitor A3K, the inter alpha-trypsin inhibitor heavy chain 4 and the serine protease inhibitor A3N were the proteins with the highest protein expression levels that were elevated in TBI mice when compared to sham (Figure S3B). We also detected a variety of proteins that have been described to be involved in inflammasome signaling, such as complement C5, serotransferrin, complement factor H, complement C4-B and complement factor B, which were elevated in the TBI group when compared to sham mice (Figure S3C). Finally, we also identified the following signaling proteins that have been shown to be involved in cardiac-related signaling events, such as ceruloplasmin, clusterin, plasminogen, kininogen-1, haptoglobin and inter alpha-trypsin inhibitor heavy chain H2, which were elevated in the TBI group when compared to the sham group (Figure S3D) [33,34,35,36,37,38].

### 2.8. Serum-Derived EVs Activate the Inflammasome in Cardiovascular Cells

**Figure 7:**
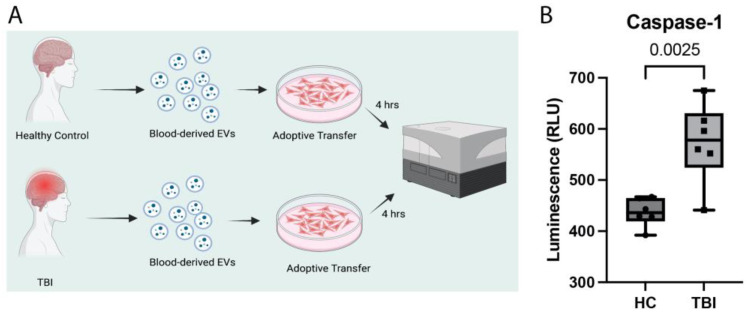
Adoptive transfer of serum-derived EVs into cardiovascular cells in culture. Experimental paradigm of adoptive transfer of serum-derived EVs from healthy control subjects and TBI patients delivered into T/G HA-VSMC human cardiovascular cells. Cells were harvested after 4 h and processed for caspase-Glo 1 activity assay (**A**). Catalytically active caspase-1 levels in the media of cardiovascular cells exposed to EVs from healthy controls and TBI patients for 4 h (**B**). Data presented as min and max. N = 6 per group. Created with BioRender.

To confirm that EVs contribute to inflammasome activation after TBI, we then isolated EVs from the serum of patients with TBI and from healthy controls and adoptively transferred the EVs to T/G HA-VSMC human cardiovascular cells, then harvested the cells after 4 h (Figure 7A), prior to analysis of caspase-1 activity in the cell media. Our findings indicate that caspase-1 activity increased in the cells that were exposed to EVs from TBI patients when compared to cells that were exposed to EVs from healthy controls (Figure 7B), indicating that the inflammasome is activated in cardiovascular cells by EVs containing inflammasome proteins after TBI.

## 3. Discussion

In this study, we show that the acute, immediate cascade of inflammatory mediators released after TBI also results in cardiac injury and significant elevation of inflammasome proteins in both the cortex and atria of the heart. Our data demonstrate that the pyrin inflammasome plays a role in the activation and processing of IL-1β in the cerebral cortex after severe TBI in mice. However, in the heart, the systemic inflammatory effects after TBI involve the upregulation of the AIM2 inflammasome in the atria. TBI resulted in the release of EVs into the serum, which contain a cargo of inflammasome-, complement- and cardiovascular-related signaling proteins, and adoptive transfer of EVs from TBI patients resulted in inflammasome activation in cardiovascular cells. Thus, TBI induces wide-ranging systemic inflammatory mediators, including inflammasomes and EVs that alter the biology of the heart.

The secondary injury cascade following TBI involves dysfunction of the autonomic nervous system (ANS), activation of the hypothalamic–pituitary–adrenal (HPA) axis, and a neuroinflammatory response [2] resulting in glucocorticoid and catecholamine release, which contribute to systemic complications [5]. Furthermore, there is a rapid depletion of adenosine triphosphate (ATP) in myocardial tissue, which is associated with myocardial band necrosis and irregular cross-band formation [22].

TBI results in the acute release of catecholamines, which may remain elevated even 14 days after injury, characteristic of the activation of the SNS [39]. This increase in catecholamines from sympathetic nerve terminals into the myocardial interstitium opens β1 adrenergic receptor-controlled calcium channels and depletes adenosine triphosphate (ATP) [32]. This process leads to cell death and myocardial contraction band necrosis, which is characterized by focal myofibrillar degeneration, myocytolysis and irregular cross-banding [32]. In our study, similar morphological changes were evident in heart sections from TBI animals at 3 days after injury, suggestive of myocardial band necrosis. However, further studies are needed to determine whether these pathological changes are due to sympathetic storming, inflammasome activation and/or a combination of distinct inflammatory mediators.

TBI results in a systemic inflammatory response syndrome (SIRS) [40] in which inflammatory proteins such as damage associated molecular patterns (DAMPs), cytokines, chemokines, coagulation factors, growth factors and nitric oxide increase in the systemic circulation, eventually causing damage to several organs [40]. Our data revealed elevated levels of IL-1 β in the cortex and ventricles of the heart after TBI, thus supporting other studies of multiple traumas in pigs, which showed IL-1 β was elevated in cardiac tissue of the left ventricle [41].

Our results showed that severe CCI induced increased levels of pyrin, caspase-1 and ASC in the cortex acutely after injury, suggesting that activation of the pyrin inflammasome plays a role in the innate immune response after injury. In addition to pyrin, other inflammasomes such as the NLRP1 and NLRP3 inflammasomes may also be activated after injury.

Pyrin plays various roles in many cellular mechanisms related to inflammatory responses. For instance, pyrin inflammasome activation influences trafficking of lymphocytes at the blood–brain interface [42]. Pyrin is encoded by *MEFV*, and mutations in *MEFV* are present in patients with familial Mediterranean fever (FMF) [43]. FMF is an autoinflammatory disease characterized by recurrent attacks and increased IL-1 synthesis. However, it is still unknown why the disease is characterized by multiple attacks. It appears that activation of the stress-induced sympathoadrenal system leads to inflammasome activation, which triggers attacks [44]. Thus, our finding that the pyrin inflammasome is activated in the cortex after TBI may be due to increased catecholamines released during the stress response, which upregulate the pyrin inflammasome. In support of this idea is the finding that exposure of human primary monocytes to phenylephrine co-stimulated with LPS increases gene expression of inflammasome proteins, increases IL-1β secretion and modulates the IL-18 response [45].

Our results showed that the AIM2 inflammasome played a role in the pathology of myocardial contraction band necrosis in that AIM2 inflammasome proteins were significantly increased in the atria of the heart after TBI. Accordingly, in the heart, we found a significant increase in the expression of AIM2 inflammasome signaling proteins, including caspase-1, ASC, caspase-1 and caspase-11 in the atria, but not the ventricles of TBI mice when compared to sham mice. Conversely, we detected increased expression of IL-1β in the ventricles of TBI mice, but not in the atria, suggesting that TBI induced inflammasome activation, and that IL-1β production occurs in the atria and then IL-1β may be secreted into the ventricles, where further pro-inflammatory events take place. Moreover, it is possible that inflammasome activation and IL-1β release from the atria may result in pyroptotic death of cells in the ventricles. Lastly, since the atrium is highly innervated by sympathetic neurons in comparison to the ventricles, it is possible that the catecholamine surge released after TBI may influence IL-1β processing and release differently in the two chambers of the heart. However, further work is needed to test this hypothesis. These results are consistent with previous studies that showed that pulmonary dysfunction following TBI involves AIM2 activation in the lungs, inducing pyroptosis and resulting in acute lung injury [46]. Thus, the pathogenesis of remote organ injury after TBI appears to be mediated by the systemic release of nucleic acid DAMPs that activate the AIM2 inflammasome in a tissue-specific manner, propagating inflammation after trauma and leading to cell death.

Importantly, here, we also found an increase in ASC specks in the brain and the heart of TBI mice, suggesting that these prion-like ASC structures play a significant role in the inflammatory pathogenesis observed after TBI in the brain and systemically. Moreover, this finding emphasizes the potential benefit of therapies targeting ASC specks after CNS injury [47].

Our earlier studies revealed that EVs derived from cerebral spinal fluid after SCI and TBI are enriched with innate immune signaling proteins that induce inflammasome activation in peripheral organs [7,18,19,28]. Here, we extended these observations using mass spectrometry analysis to demonstrate that serum EVs after TBI contain myriad inflammasome-, complement- and cardiovascular-related signaling proteins. Some of the proteins found in EVs have been implicated in inflammasome signaling such as the complement C3 [48] and C5 proteins [34], while others are also involved in cardiovascular-related events like ceruloplasmin [37], clusterin [33], plasminogen [35], kininogen [38] or haptoglobin [36]. In particular, the complement component C3 was abundantly present in serum-derived EVs after TBI. Complement components have been demonstrated to increase in CSF and in blood plasma in TBI patients [49] and the complement components C3 and C9 are elevated up to 6 months post-injury [50], suggesting continuous complement pathway activation in chronic TBI. Deposits of C3 have also been described in perilesional tissue after cortical injury in the adult rat [51]. Thus, the complement–inflammasome axis plays an integral part in the pathogenesis of acute and chronic inflammatory conditions [52,53]. However, the mechanism by which EVs containing inflammasome-, complement- and cardiovascular-related signaling proteins influence the systemic inflammatory response remains to be determined.

Cardiovascular complications occur after TBI, which may lead to increased morbidity and mortality [21] due to hypotension, hypertension, ECG changes, cardiac arrhythmias, release of heart injury markers or left ventricular (LV) dysfunction [2,32]. Often, the severity of these cardiovascular complications correlates with TBI severity [54]. One clinical study reported elevated levels of catecholamines in necrotic cardiac lesions of motor vehicle accident patients who suffered from diffuse cerebral injury [30]. Another study found an association between the history of TBI and TBI severity, with altered cardiovascular autonomic regulation compared to patients with no history of TBI [55]. TBI patients may present with reduced diastolic capacity, arrhythmias and other electrocardiogram changes [31,32,56]. Elevated rates of isovolumic relaxation time and higher apical strain in TBI patients compared to controls [31] have been measured using echocardiography. Additionally, our previous studies have shown that inflammasome signaling proteins are useful biomarkers of TBI [46,57,58,59]. Our current findings, indicating that adoptive transfer of EVs from TBI patients into cardiovascular cells activates the inflammasome, reinforce our previous work showing that EVs released after TBI also trigger an inflammatory response consistent with acute lung injury [7,28,29]. In addition, the presence of an inflammatory response in the heart after TBI, mediated by the inflammasome, highlights the role of the innate immune response after CNS injury in the cardiovascular system and is consistent with previous studies showing that inflammasome activation in the heart is associated with heart failure [60] and cardiac hypertrophy [61].

Despite histological and inflammatory changes in the heart 3 days after TBI, in this study, we did not assess for cardiovascular function. Future studies should aim to characterize cardiac function after TBI, including the effects of inflammasome inhibition after TBI in the cardiovascular system. Moreover, here, we only analyzed tissues at 3 days after injury due to the acute phase of the innate immune response associated with the inflammasome. Future projects will study the effects of TBI in the heart at more chronic time points to determine the long-term consequence of TBI in the cardiovascular system.

Our data support the idea that the pathophysiology of TBI is highly complex and heterogeneous and that secondary injury mechanisms regulating cardiac pathology also include EV-mediated AIM2 and pyrin inflammasome activation. Therefore, targeting the neuronal–cardiac–inflammasome axis by modulating inflammasome activation may provide a novel therapeutic approach for the treatment of neurogenic cardiac injury.

## 4. Materials and Methods

### 4.1. Animals and TBI

All animal procedures were approved by the Animal Care and Use Committee of the University of Miami (protocol 19–164). Animal procedures were carried out according to the Guide for the Care and Use of Laboratory Animals (U.S. Public Health, Washington, DC, USA). C57BL/6 male mice (30 g, ~11 to 15 weeks-old) (The Jackson Laboratory, Bar Harbor, ME, USA) underwent controlled cortical impact (CCI) brain injury, as described in [7]. Animals were randomly assigned so that on surgery days, similar numbers of sham and TBI animals were generated. Briefly, a 5 mm craniotomy was performed in the right parietotemporal cortex without disturbing the dura. The epicenter of injury was at −2.0 mm from bregma and 2.5 mm laterally. Animals were then transferred to a stereotaxic frame in a holder for cortical contusion injury with the 68099II Precise Impactor (RWD). Animals underwent CCI at a velocity of 5.5 m/s and a depth of 0.5 mm. Animals were sacrificed 3 days after CCI. The brain cortex and heart of each animal were then removed, and protein lysates were obtained for the cortex and the heart. Each heart was dissected into atria and ventricles. Sham uninjured animals were used as controls. Lysed protein samples were then stored at −80 °C until analyses. Serum samples were stored at −80 °C until processing for EV isolation. Similarly, a group of R26-CAG-ASC-citrine mice (B6.Cg-Gt(ROSA)26Sor^tm1.1(CAG-Pycard/mCitrine*,-CD2*)Dtg^/J, The Jackson Laboratory, Bar Harbor, ME, USA) was used for imaging of ASC specks in the brain and heart after TBI. Sham animals underwent all surgical procedures except TBI.

### 4.2. Perfusion/fixation and Immunohistochemistry

Animals underwent perfusion–fixation, as in [11]. A median sternotomy was then performed, and the intact heart was dissected and post-perfused for 72 h in 4% PFA. Heart tissue was then paraffin embedded prior to sectioning at 15 μm. Sections were stained for hematoxylin and eosin (H&E). In addition, frozen brain and heart sections from R26-CAG-ASC-citrine mice were processed for confocal microscopy and cover-slipped using VECTASHIELD^®^ Vibrance with DAPI, then imaged in the Andor Dragonfly Confocal Imaging System, Model 200 (Oxford Instruments, Abington, Oxfordshire, UK).

### 4.3. IL-1β Protein Analysis Assay (ECLIA)

IL-1β levels in protein lysates from the brain cortex and heart of sham and TBI mice were determined, as described in [14], using the MESO Quickplex SQ 120 instrument, Rockville, MD, USA.

### 4.4. Immunoblotting

Brain protein lysates were obtained, as described in [62,63], and 25 μg of protein lysates was then resolved via immunoblotting for the expression of inflammasome signaling proteins, as described in [64]. Primary antibodies used in this study were against the following proteins: NLRP1 (Novus Biologicals, Centennial, CO, USA), NLRP3 (Novus Biologicals Centennial, CO, USA), AIM2 (eBioscience, San Diego, CA, USA), NLRC4 (Novus Biologicals, Centennial, CO, USA), pyrin (Santa Cruz Biotechnology, Inc. Dallas, TX, USA), caspase-1 (Novus Biologicals, Centennial, CO, USA), ASC (Santa Cruz), IL-1β (Cell Signaling, Technology, Inc. Danvers, MA, USA), caspase-8 (Novus Biologicals, Centennial, CO, USA), caspase-11 (Novus Biologicals, Centennial, CO, USA), CD63 (Novus Biologicals), β-actin (Sigma-Aldrich, St. Louis, MO, USA) and GAPDH (Sigma Aldrich, St. Louis, MO, USA).

### 4.5. Partial Purification of ASC Specks

ASC specks were partially purified, as in [65].

### 4.6. Extracellular Vesicle (EV) Isolation

EVs were isolated from serum using the Total Exosome Isolation Reagent (Invitrogen, Carlsbad, CA, USA), as described in [8]. Briefly, 100 μL of serum was mixed with 20 μL of Total Exosome Isolation Reagent (from serum). Samples were then incubated for 30 min and centrifuged at 10,000× *g* for 10 min. Following that, the supernatant was discarded and the pellet containing the EV resuspended. For adoptive transfer of EVs, samples were resuspended with phosphate-buffered saline (PBS), and for the Ella Simple Plex Assay (below), samples were resuspended in protein lysis buffer.

### 4.7. Inflammasome Protein Characterization in EVs with the Simple Plex Assay

To characterize inflammasome proteins in EVs from humans after TBI, 200 μL of serum from 16 TBI patients (25 to 68 years old) and 16 healthy controls (15 to 78 years old) was used to isolate EVs, as described above. Samples were collected at Son Espases University Hospital (Palma de Mallorca, Spain) (IRB: 3127/15). Written informed consent was obtained, as in [66]. EV proteins were extracted with lysis buffer containing protease inhibitor cocktail (Sigma), and 50 μL of diluted sample (2-fold dilution) was run in the Ella Simple Plex (Protein Simple), as described in [67].

### 4.8. Nanoparticle Tracking Analysis (NTA)

Isolated EVs were analyzed for particle size and particle concentration with the NanoSight NS300 instrument (Malvern Panalytical, Ltd., Malvern, UK) using Nanosight NTA 2.3 software Malvern Panalytical, Ltd., Malvern, UK), as in [7,19,28]. Briefly, 2 μL of EVs was added to 998 μL of distilled (DI) water to prepare the NTA sample. The instrument was first flushed with approximately 3 mL of DI water. Approximately 300 μL of sample was loaded into the NanoSight NS300 for analysis.

### 4.9. Mass Spectrometry

To analyze the protein composition of EVs isolated from the serum, EVs were isolated and analyzed with mass spectrometry by Northwestern Proteomics. Briefly, samples were sonicated for lysis, centrifuged at 10,000× *g* for 10 min at 4 °C to remove cell debris, and the supernatant containing the EV proteins was collected. Collected samples were then purified with trichloroacetic acid (TCA) precipitation, and after a stacking gel method, the whole sample was then cut out as one band. Then, proteins were in-gel digested and processed, as in [68]. MS/MS spectra were searched against the SwissProt *Mus musculus* database with the Mascot search engine (Matrix Science, London, UK; version 2.7.0.1). Searches consisted of carbamidomethyl Cys as a fixed modification and oxidized Met, deamidated Asn and Gln and acetylated N-term as variable modifications. Results were visualized using Scaffold software version 4 (Proteome Software, Portland, OR, USA).

### 4.10. Adoptive Transfer of EVs into Cardiovascular Cells

T/G HA-VSMC human cardiovascular cells (American Type Culture Collection (ATCC), Manassas, VA USA) were grown in culture to 70% confluency in F12K media containing fetal bovine serum, exosome-depleted, One Shot™ format (Gibco, Grand Island, NY, USA). EVs were isolated from the serum of 6 TBI patients (38 to 83 years old) (patient characteristics in Table S1) and 6 healthy controls (38 to 56 years old), as described. Cells were harvested at 4 h after EV exposure (~4.3 × 10^7^ particles/mL), and the cell medium was then analyzed using the Caspase-Glo^®^ 1 Inflammasome Assay (Promega, Madison, WI, USA) according to manufacturer’s instructions in a white 96-well plate, and luminometry was performed in the SPARLK 10M (TECAN) spectrophotometer, Seestrasse, Switzerland.

### 4.11. Statistical Analyses

Prism 9.0 software (GraphPad Software, San Diego, CA, USA) was used for statistical analyses. Normality was determined using the Shapiro–Wilk test. Statistical comparisons between two groups were conducted using Student’s *t*-test for parametric data or the Mann–Whitney test for non-parametric data. The P-value of significance used was <0.05. All outcome measures were evaluated by investigators who were blinded to the experimental groups.

## Data Availability

Available data will be provided upon request to the corresponding author.

## Supplementary Materials

The following supporting information can be downloaded at: https://www.mdpi.com/article/10.3390/ph16101382/s1, Figure S1: Inflammasome sensor protein expression in the cortex of mice. Figure S2: Characterization of serum-derived EVs from TBI patients. Figure S3: EV characterization with MS. Table S1: TBI patient characteristics.
