# Supplementary material for: Neural–Cardiac Inflammasome Axis after Traumatic Brain Injury
Source: Pharmaceuticals (Basel). 2023 Sep 28;16(10):1382. doi: 10.3390/ph16101382 (PMC10610322; doi:10.3390/ph16101382)
Supplement: Supplementary file 1 [file pharmaceuticals-16-01382-s001.zip › pharmaceuticals-2564836-supplementary.pdf]

pharmaceuticals

Supplementary Materials

Neural–Cardiac Inflammasome Axis after Traumatic Brain Injury

Robert W. Keane, Roey Hadad, Xavier O. Scott, Erika d. l. R. M. Cabrera Ranaldi, Jon Pérez-Bárcena and Juan Pablo de Rivero Vaccari

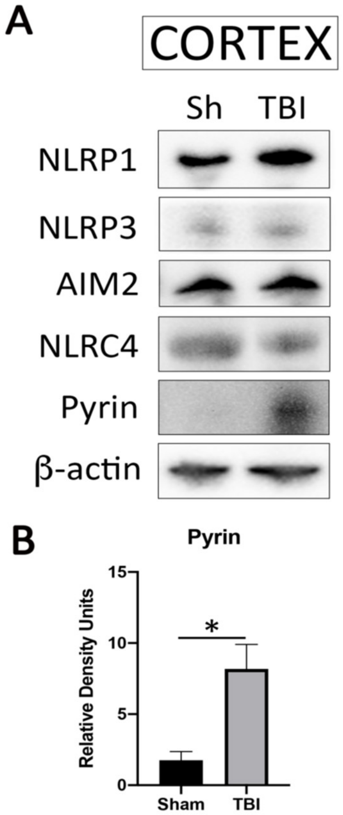

Figure S1. Inflammasome sensor protein expression in the cortex of mice. (**A**) Immunoblot of cortical protein lysates of.sham and TBI mice that were injured and sacrifice 3 days after trauma and blotted for the inflammasome sensor protein NLRP1, NLRP3, AIM2, NLRC4 and pyrin. (**B**) Quantification of pyrin in sham and TBI mice. β-actin was used as a protein loading control and internal standard. Data presented as mean + SEM. N = 7 per group. * $p < 0.05$.

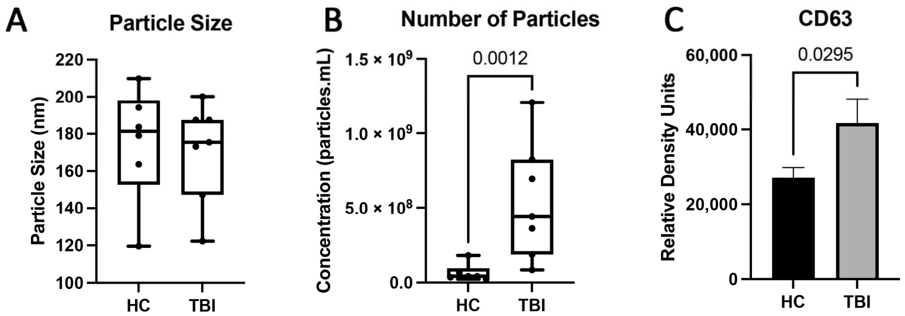

Figure S2. Characterization of serum-derived EV from TBI patients. NTA results for particle size (**A**) and particle number (**B**) of healthy controls (HC) and TBI patients. N: HC: N = 6 and TBI: N = 7.

(**C**) Relative protein expression levels of the EV marker CD63 in EV isolated from the serum of HC and TBI patients. N = 7 per group.

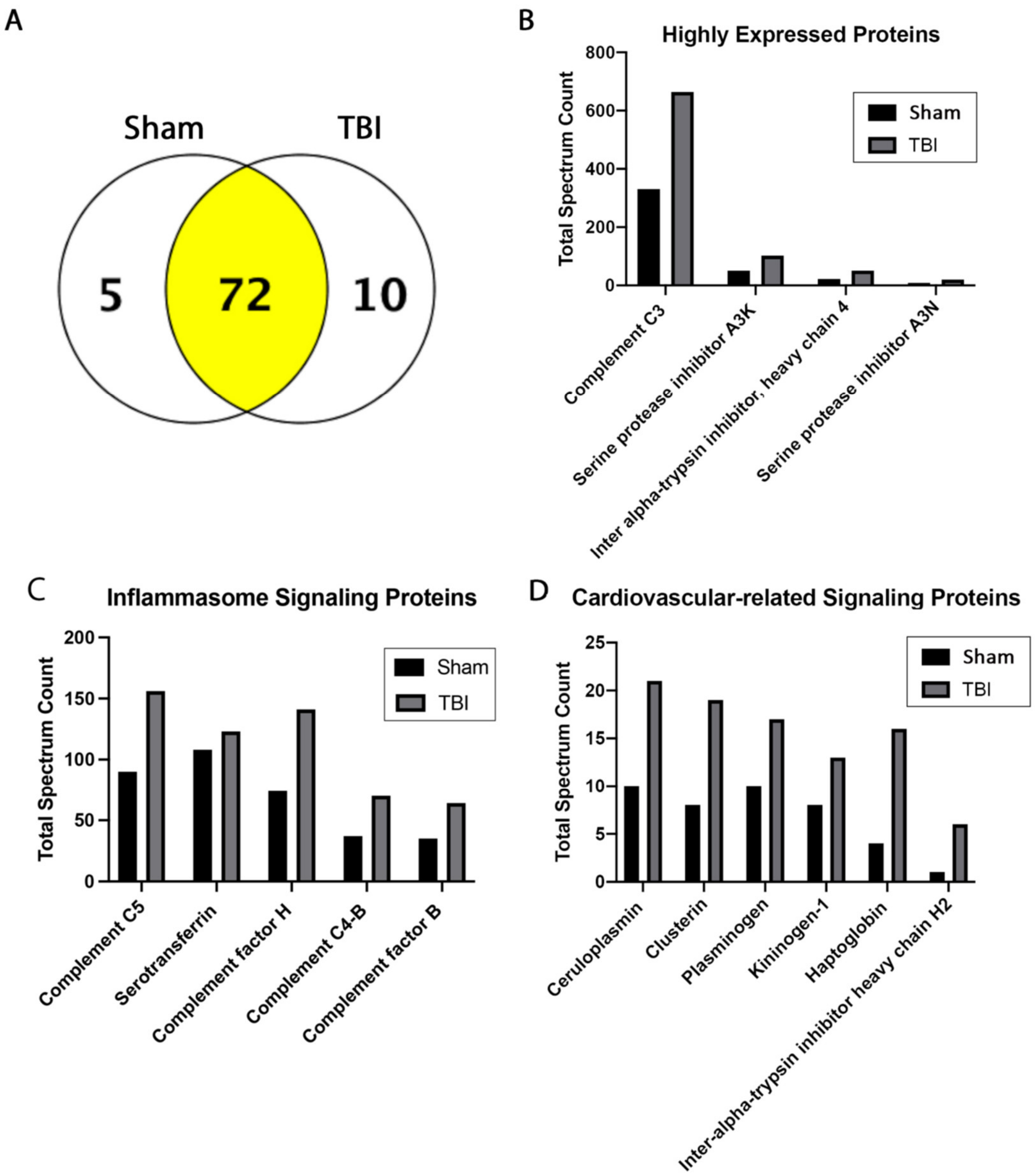

Figure S3. EV characterization by MS. (**A**) Venn diagram of the protein distribution between sham and TBI mice. (**B**) Bar graph of proteins with the highest expression in EV. (**C**) Bar graph of proteins known to be involved in inflammasome signaling that are elevated in EV from TBI mice when compared to sham. (**D**) Bar graph of proteins known to be involved in cardiac related signaling events that are elevated in EV from TBI mice when compared to sham.

Table S1. TBI Patient Characteristics.

Hospital Stay		Epidemiology		
Days in Hospital	Days in ICU	Age	Sex	Type of Accident
**2**	**1**	58	1	MVA
5	5	63	1	Fall
9	1	49	0	Fall
23	23	39	1	Fall
25	25	38	1	Assault

12	3	83	1		Fall

**Comorbidities**			**Brain Trauma**					
**Hypertension**	**Diabetes**	**Cerebral Ischemia**	**GCS**	**GCS M**	**GCS V**	**GCS O**	**Pupillary Reactivity**	**Cranial Surgery**
0	0	0	12	6	3	3	2	0
0	1	0	8	5	2	1	2	1
0	0	0	14	6	5	3	2	1
0	0	0	5	3	1	1	2	1
0	0	0	3	1	1	1	2	1
1	1	1	15	6	5	4	2	0

**Characteristics @ Admission**					
**Hypoxia**	**Hypotension**	**Subarachnoid Hemorrhage on CT**	**Epidural Mass on CT**	**Glucose**	**Hb**
0	0	1	0	128	14.3
0	0	1	0	472	14.3
0	0	0	1	112	11.7
1	1	1	0	260	12.5
0	0	0	0	153	14.5
0	0	1	0	140	11.6

**Subdural Hematoma**	**Contusion**	**GOSE**
1	0	8
1	1	1
1	0	8
0	1	1
1	1	4
1	0	5

Definitions	
0	No
1	Yes
GCS	Glasgow Comma Scale
GCS M	Motor
GCS V	Verbal
GCS O	Ocular
Pupils (2)	Both pupils react
Hb	Hemogloblin
GOSE	Glasgow Outcome Scale Extended
